# SCiPad: Effective Implementation of Telemedicine Using iPads with Individuals with Spinal Cord Injuries, a Case Series

**DOI:** 10.3389/fmed.2017.00058

**Published:** 2017-05-29

**Authors:** Kazuko Shem, Samantha J. Sechrist, Eleanor Loomis, Linda Isaac

**Affiliations:** ^1^Department of Physical Medicine and Rehabilitation, Santa Clara Valley Medical Center, San Jose, CA, United States; ^2^Division of Physical Medicine and Rehabilitation, Department of Orthopaedic Surgery, Stanford University, Stanford, CA, United States; ^3^Rehabilitation Research Center, Santa Clara Valley Medical Center, San Jose, CA, United States; ^4^Physical Medicine and Rehabilitation, Occupational Medicine, The Permanente Medical Group Inc., Sacramento, CA, United States

**Keywords:** spinal cord injury, telemedicine, quality of life, telehealth, community reintegration, secondary complications

## Abstract

**Background:**

Individuals with spinal cord injury (SCI) must often travel long distances to see a rehabilitation specialist. While telemedicine (TM) for pressure ulcer management has been used in this population, real-time video telecommunication using iPad has never been described.

**Objective:**

The objective of this study was to provide specialized care for persons with SCI through TM consultation expediently in order to address medical needs, manage secondary complications, and to improve quality of life (QoL) of individuals with SCI.

**Methods:**

Ten individuals with SCI participated in the TM program using iPads for 6 months as a feasibility study at a single-center, county hospital. The participants contacted the project staff for SCI-related conditions and were then connected to an SCI-trained health-care provider within 24 hours via FaceTime. Main outcome measures included health-care utilization; QoL and psychosocial measures collected at baseline and at 6 months: Reintegration to Normal Living Index (RNLI), Life Satisfaction Index A (LSI-A), and Patient Health Questionnaire 9 (PHQ-9); and a Program Satisfaction Survey.

**Results:**

Ten patients (seven with tetraplegia, three with paraplegia; eight males and two females) with an average age of 34.4 (18–54) years were enrolled. The average baseline and 6-month follow-up scores were RNLI—70.1 ± 19.7 and 74.7 ± 21.8, respectively; LSI-A—25.4 ± 7.4 and 26.4 ± 8.2, respectively; and PHQ-9 were 6.8 ± 7.2 and 8.6 ± 6.1, respectively. TM encounters included topics such as pain, bladder and skin management, medication changes, and lab results. The Program Satisfaction Survey yielded positive results with 100% of program completers stating they would recommend the program and would like to continue having TM.

**Conclusion:**

This is the first known successful project using iPad to provide TM in the SCI population. This study discusses the implementation of such a TM program in a health system including limitations. It describes the clinical viability of TM using iPads in the SCI population for care beyond that of just pressure ulcer management. This project provides evidence for using a tablet device like an iPad as an effective and efficient patient management tool.

## Introduction

Spinal cord injury (SCI) is a devastating injury that not only causes paralysis but also causes significant chronic morbidity due to secondary complications. There are an estimated 282,000 people living with SCI in the United States (1). Individuals with SCI face many challenges upon transition from acute inpatient rehabilitation. Due to their medical complexity and high risk for secondary complications, they often require rehospitalizations and regular ongoing medical and psychological care for a variety of issues (2–6). Furthermore, many of these patients, as is true in general for persons with disabilities, live in rural areas without immediate medical care or rehabilitation specialists and must travel hours to see an SCI specialist (7). Unfortunately, this can lead to delays in diagnosis and treatment of secondary complications and may ultimately impede the patient’s ability to reintegrate back into their communities and lead productive lives.

One method in which to provide care to these individuals is through telemedicine (TM). TM is an application of technologies to enable accessibility of medical services. These tools allow for low-cost and wide-reaching solutions in providing specialized consultation expediently to those persons with SCI who live in rural areas. Furthermore, TM helps eliminate geographical barriers and can improve patient access to medical services that often would not be consistently available in distant rural communities. To date, there are limited studies focusing on delivery of care via telehealth to the SCI population specifically (8).

In the field of SCI, there are a number of studies that give support to the treatment of pressure sores through the use of TM, which allows for both maintenance of strict bed rest and visualization of the skin by an SCI specialist with the goal of faster resolution of the pressure sores (9, 10). Smith et al. conducted a modeled analysis of the telehealth cost implications for treatment or prevention of pressure ulcers and found that telehealth care was less costly compared to standard of care when low-cost technology (digital cameras and e-mail) was used. Additionally, increased use of telehealth could reduce the occurrence of stage III and IV ulcers; therefore, quality care was provided while preventing costs (11).

A study of Dallolio et al. showed that satisfaction with care was improved in individuals with SCI who received TM interventions (12), and a large randomized control trial showed that TM interventions in individuals with SCI may decrease rates of rehospitalization after hospital discharge (13). Some telehealth programs require patients to travel to a local health center for communication with another, more specialized, center of care. Although care is not provided in the home setting in this case, it still allows for specialized treatment to be given more locally. Even given these benefits and the existence of telehealth for decades, TM is still not standard of care for the SCI population.

Currently, the development of telehealth clinical protocols and the exploration of adaptive devices that can accommodate the functional limitations of persons with SCI are lacking (8). Given the difficulties persons with SCI may face to travel to and from clinics or even videoconferencing centers, a potentially more convenient technological solution was sought after. We are not aware of any TM program using iPads for patient–provider communication in the SCI patient population. The use of iPads facilitates communication within the home rather than requiring travel to a satellite center and provides a user-friendly interface.

This proof of concept study provided a live interactive TM consultation in which the provider with expertise in SCI and the patients were connected via FaceTime on an iPad. The iPad was chosen as the TM device given its established ease of use for videoconferencing without additional equipment, its portability, and its ease of use for patients with physical limitations including impaired hand function. FaceTime was selected as it was the most secure mode of telecommunication with appropriate encryption that was available at the time of initiation of this project. The objectives of this project were to address patients’ needs expediently, to provide more health-care encounter opportunities with an SCI specialist, to treat and address secondary complications of SCI and thereby prevent the need for SCI patients to seek emergency care and potential hospitalizations, to decrease the need for SCI patients to seek care from non-SCI specialists, and to improve quality of life (QoL) in individuals with SCI. Importantly, this proof of concept project was designed to assess the feasibility of TM using iPads in the SCI population and subsequently extend the reach of the SCI specialty care and expertise provided by a rehabilitation center with SCI focus.

## Materials and Methods

### Setting

This was a single-center study conducted by a rehabilitation service at a county hospital. This study enrolled 10 participants between May and June 2014. This study was carried out in accordance with the approval of the Institutional Review Board’s (IRB’s) Research and Human Subjects Review Committee of Santa Clara Valley Medical Center. All participants understood and gave written consent in accordance with the Declaration of Helsinki.

### Participants

Participants were included if they were 18 years or older at the time of enrollment and had a traumatic or non-traumatic SCI at any neurological level. Participants were prioritized for enrollment if they were being discharged from the acute inpatient rehabilitation program at the study institution since these patients often require more support soon after their discharge from the hospital. Participants were excluded if they had inadequate command of the English language as the health-care providers interacting with the participants were strictly English-speaking. Participants were also excluded if they lived outside of the state of California and/or if they had insurance that did not approve TM visits with the study provider. All participants included in the study completed the informed consent forms and the Health Insurance Portability and Accountability waiver.

### Program Implementation

Due to the size of our facility’s catchment area for acute inpatient rehabilitation, patients often travel hours to come for in-person visits with an SCI specialist. Oftentimes, these patients are unable to find specialized care in remote areas or are not able to return to our facility due to transportation issues after discharge. For decades, our outpatient SCI physicians have traveled to see these patients in rural areas to conduct outpatient clinics in Northern and Central California approximately 8–10 times per year. Due to these challenges, the SCiPad TM program was created in an effort to provide more health-care opportunities with an SCI specialist and explore the feasibility of using iPads to deliver this care.

To establish this TM program, we first obtained funding from the Craig H. Neilsen Foundation for 1 year. Next, we obtained approval from our hospital administration including authorization from the chief medical officer, chief information officer, compliance office, research administration, and the IRB prior to initiating the program. We reviewed security issues with the data plan vendor (Verizon) and confirmed that the data plan would be double encrypted. The last step before providing our first TM visit was that we also needed to coordinate with our Electronic Medical Record department, billing service, and Scheduling Maintenance service so that we can schedule visits in our system as “TM” visits and document and bill the visits appropriately.

### Technical Devices and Adaptive Equipment

All participants received an Apple iPad Air which they were allowed to keep at the end of the study. Upon receiving the iPad, the program coordinator made sure that each participant had a password-protected Apple ID, which the care provider would use as the contact for TM visits. Occupational therapists (OTs) were consulted for the specific needs for each participant. Patients with higher level of injuries (C1–C4) with limited upper extremity functioning were provided with either a mouthstick with a capacitive stylus (such as Pogo or BoxWave) or a TouchTec Multi-Function Capacitive Touch Screen Mouthstick if recommended by an OT. Additional accessories included a mounting bracket with rotating arm to mount the iPad on a wheelchair. Individuals with a C5–C7 level of injury may use an adapted stylus such as the Steady Stylus or a capacitive stylus (BoxWave or Pogo) with u-cuff or other hand splint if recommended by an OT. The participants were provided with 6 months of cellular data plan with Verizon Wireless, and they were informed that we had the capability of monitoring their data usage, *via* MaaS360, if there were any concerns during the 6-month period of participation. If necessary, MaaS360 allowed us to remotely locate, lock, and wipe lost or stolen devices. Moreover, the wireless connection was protected by double encryption to provide confidentiality.

### Procedures

#### Data Collection

Patients were followed for 6 months and were contacted monthly by the program coordinator to complete follow-up interviews regarding demographic updates, health-care utilization, and medical complications. At baseline and during the 6-month follow-up, patients also completed questionnaires regarding QoL. Additionally at 6 months, participants were asked to complete a Program Satisfaction Survey.

#### TM Encounters

Participants were to contact the program coordinator to schedule a TM appointment as needs arose. Participants were not restricted from seeking out other in-person medical care. For emergency situations, participants were instructed to seek emergency care from their local emergency room (ER); although we aimed to provide expedited care, the TM appointments were not meant to be relied on in an urgent, life-threatening emergency situation.

**Figure 1:**
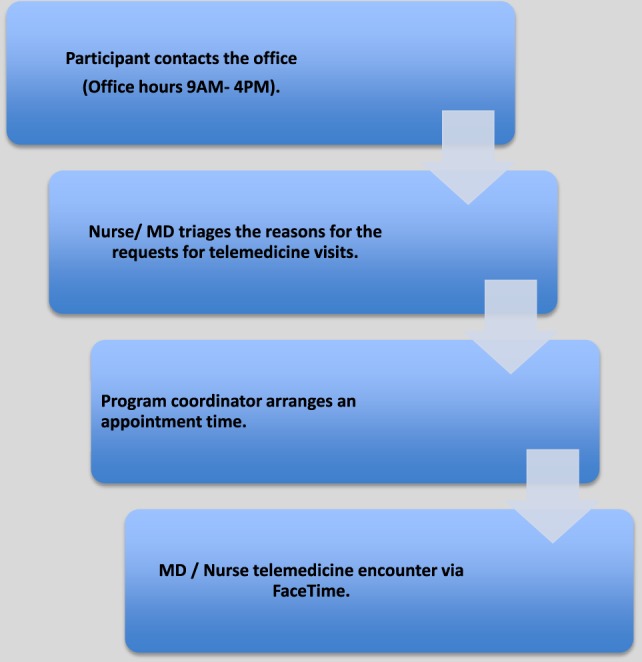
**Telemedicine encounter flow diagram**.

For TM appointments, study participants were instructed to contact the study coordinator between 9:00 a.m. to 4:00 p.m. on weekdays for non-emergency needs (Figure 1). Patients were then scheduled for a TM encounter regarding any questions related to their SCI conditions including but not limited to bowel and bladder management, pressure ulcers, spasticity, pain, depression, equipment, therapies, and prescriptions. Encounters with an SCI specialist could be setup within 24 hours on weekdays during office hours, if necessary.

Spinal cord injury specialists included an SCI physiatrist or registered nurse with experience in SCI issues. Encounters with a nurse were monitored by the principal investigator who provided additional assistance if the issues were beyond the scope of practice of the nurse. All TM encounters were documented in the patient’s medical record and were reviewed for data collection.

### Measures

#### Patient Characteristics

Upon enrollment, demographic and injury-related information such as age, gender, ethnicity, level of injury, etiology of SCI, and concomitant traumatic brain injury were collected from each participant (Table 1).

**Table 1:** **Patient (*n* = 10) characteristics**.

Characteristics	Value
**Enrollment**	
From inpatient	8
From outpatient	2
**Sex**	
Male	8
Female	2
Age at enrollment	34.4 ± 14.4
**Marital status at enrollment**	
Single	4
Married	2
Divorced	2
Separated	1
Widowed	1
**Ethnicity**	
Caucasian	6
Hispanic	4
**Etiology**	
Motor vehicle accident	5
Gunshot wound	2
Others	3
**Level of injury**	
Cervical	7
Thoracic	3
**AIS**	
A (complete)	7
B (sensory incomplete)	1
C (motor incomplete)	2
**Concomitant traumatic brain injury**	5

*Values are n or mean ± SD*.

#### Health-care Utilization

Participants were contacted monthly by the program coordinator to collect demographic updates (changes in marital status, employment status, and living situation), health-care utilization data (documentation of ER visits, hospitalizations, in-person physician visits, and TM encounters), and other medical data (secondary complications, changes in medications, and changes in bowel and bladder management).

#### Quality of Life

Participants were contacted at baseline and at the end of their 6-month participation for QoL and psychosocial issues using the Reintegration to Normal Living Index (RNLI), Life Satisfaction Index A (LSI-A), and Patient Health Questionnaire 9 (PHQ-9) to measure changes in community reintegration, life satisfaction, and depression, respectively.

The RNLI is an 11-item measure which captures community reintegration including topics such as mobility, self-care, daily activities, recreational activity, family roles, and level of comfort in social situations (14). This measure was scored using a visual analog scale anchored by whether or not the item describes the participant’s situation. Total scores are proportionally adjusted to a maximum of 100 with higher scores indicating greater integration (15). The RNLI is a valid and reliable measure of community participation for persons with SCI (α = 0.87) (16).

The LSI-A was designed to measure zest, resolution and fortitude, congruence between desired and achieved goals, positive self-concept, and mood tone (17). There are 20 questions, of which 12 are positive and eight negative. Responses are in an agree/disagree/undecided format. Scores range on a scale of 0–40 with higher scores indicating greater life satisfaction. The LSI-A was developed for use in studying the elderly (18); however, it has been used to examine correlates of life satisfaction and SCI (19).

The PHQ-9 is a self-reported 9-item scale that assesses the nine Diagnostic and Statistical Manual of Mental Disorders depression symptoms for frequency over the past 2 weeks. Scores are totaled on a scale from 0 to 27 and are categorized into level of severity with higher scores indicating greater depressive symptoms (1–4 = minimal depression, 5–9 = mild depression, 10–14 = moderate depression, 15–19 = moderately severe depression, and 20–27 = severe depression) (20). The PHQ-9 is a reliable measure for persons with SCI (α = 0.87) (5).

#### Program Satisfaction

At the end of the 6 months, participants completed a Program Satisfaction Survey to capture satisfaction with the overall program, experience with TM, and use of the iPad and FaceTime specifically. Anecdotal feedback was collected as well.

### Analyses

Health-care utilization variables were analyzed for amount of incidences over the 6-month period. For all QoL measures (RNLI, LSI-A, and PHQ-9), the baseline and 6-month average total scores were calculated and paired *t*-tests were used to assess for any significant changes from baseline to 6 months.

## Results

### Patient Characteristics

Ten participants were enrolled between May and June 2014 (Table 1). Eight participants were enrolled from inpatient rehabilitation and two were patients from outpatient services with older injuries. There were eight males and two female participants with an average age of 34.4 (±14.4) at the time of enrollment. The etiologies of injury are as follows: five due to motor vehicle accidents, two due to gunshot wounds, and three due to others. Seven participants had tetraplegia and three had paraplegia. Seven had a complete SCI while three had an incomplete injury. Even with a small sample size of 10, our patient demographics match that of the general traumatic SCI population historically with a gender proportion of approximately 80% male, an average age of 30–40 at the time of injury, and with the most common cause of SCI being automobile accidents (1, 21, 22).

### Health-care Utilization

**Figure 2:**
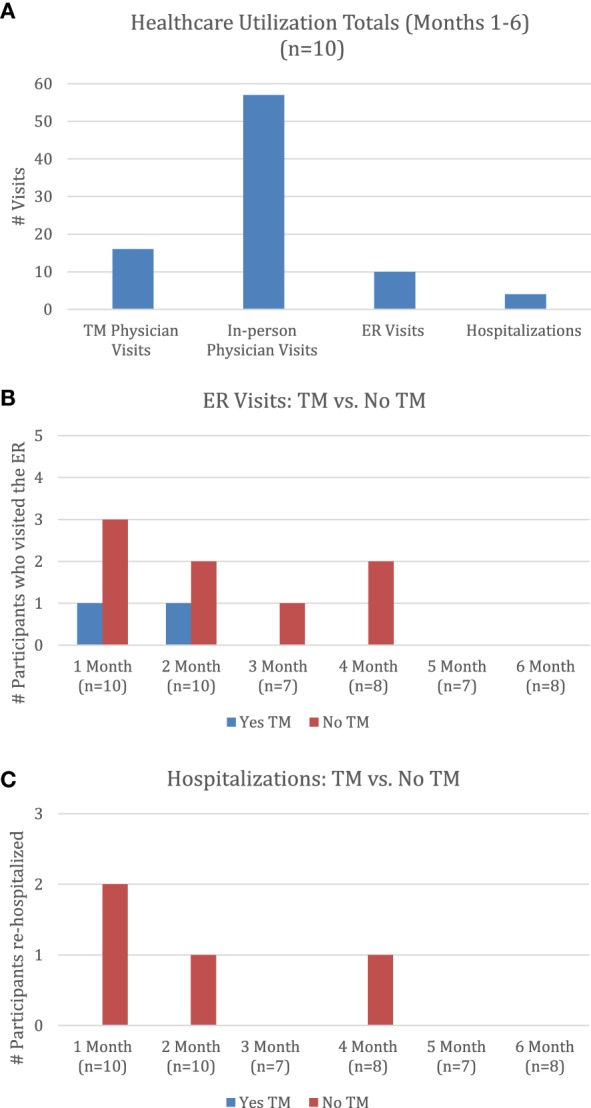
**Health-care utilization**. **(A)** Health-care utilization totals (months 1–6) (*n* = 10). **(B)** ER visits: TM vs. no TM. **(C)** Hospitalizations: TM vs. no TM. Note: months 3–6 are missing 2–3 participants’ responses. Abbreviations: TM, telemedicine; ER emergency room.

The total number of in-person physician visits, ER visits, hospitalizations, and physician TM visits were calculated (Figure 2A). Over the course of the 6-month period, 57 in-person physician visits were reported. The specialties reported from in-person visits included gastroenterology, neurology, ophthalmology, orthopedics, otolaryngology, pain management, primary care, pulmonary, urology, and wound care. A total of 10 ER visits and 4 hospitalizations occurred; the majority of the ER visits and hospitalizations were endorsed by participants who did not utilize TM on that given month (Figures 2B,C). A total of 16 TM visits occurred via FaceTime; the physician was able to successfully address topics such as spasticity, skin management, bladder and bowel management, pain, medications, heterotopic ossification, and general comprehensive follow-ups via TM. Patients were also able to receive care from a nurse either over the phone or FaceTime depending on the situation; a total of nine nurse encounters were documented throughout the study with topics ranging from skin checks, bladder irrigation, bowel training programs, and changes in urine.

Half of the participants did not utilize TM during the 6-month study. In an effort to capture qualitative data, we documented patient self-reported comments regarding reasons for not having TM (Table 2, S). Information retrieved from the Program Satisfaction Survey revealed that participants who did not utilize TM were not newly injured, were more medically stable, and did not have secondary complications to manage; one person felt more comfortable continuing to see a local physician in-person, and another patient was able to coordinate appointment times with us in-person for days when he/she had to travel near our facility for other reasons (Table 3, S).

**Table 2:** **Program Satisfaction Survey (*n* = 8)**.

	Strongly agree	Agree	Slightly agree	Neither	Slightly disagree	Disagree	Strongly disagree	NA
(A) The training I received helped me to understand how to operate my iPad.	4 (50)	2 (25)	1 (12.5)	1 (12.5)				
(B) The iPad was easy to use.	6 (75)	2 (25)						
(C) Since receiving the iPad, I have been motivated to monitor my health.	4 (50)	3 (37.5)		1 (12.5)				
(D) I feel my health has improved because of the TM program.	5 (62.5)	1 (12.5)		1 (12.5)	1 (12.5)			
(E) I was satisfied with the quality of the visual image and audio sound during my TM visit(s).	4 (50)	2 (25)						2 (25)
(F) The iPad took too much time to use.					1 (12.5)	1 (12.5)	6 (75)	
(G) I was worried about my privacy with the iPad.				1 (12.5)	1 (12.5)	2 (25)	4 (50)	
(H) The care I received through TM was just as good as seeing my physician or nurse.	3 (37.5)	2 (25)	1 (12.5)					2 (25)
(I) I would recommend the TM program.	6 (75)	2 (25)						
(J) I am satisfied with my use of the iPad.	6 (75)	2 (25)						
(K) The adaptive equipment I received was sufficient for my needs.		2 (25)						6 (75)
(L) I would like to continue to have TM visits with my physician or nurse.	4 (50)	2 (25)						2 (25)
(M) Staff responded to my needs sufficiently.	5 (62.5)	2 (25)						1 (12.5)

	**Daily**	**Several times/week**	**Once/week**	**Less than once/week**				

(N) How frequently did you use your iPad.	7 (87.5)	1 (12.5)						

	**Videos/movies**	**Games**	**Internet**	**E-mail**	**TM**	**Music**	**Other**	

(O) What purpose did you use your iPad for? *most frequent purpose shown here only*	2 (25)	1 (12.5)	4 (50)			1 (12.5)		

	**Family**	**Caregiver**	**Friend**	**Other**	**None**			

(P) Did someone other than you use the iPad?	3 (37.5)	1 (12.5)		1 (12.5)	3 (37.5)			
	Yes	No						
(Q) Do you have Wi-Fi at home?	6 (75)	2 (25)						
	**TM**	**In-person**	**Phone**	**NA**				
(R) Top preference for medical care	3 (37.5)	2 (25)	1 (12.5)	2 (25)				

	**Anecdotal comments**							

(S) If you did not have a TM visit with an SCVMC physician, please let us know why?	“I only had one TM visit. I would have had more but I was always in San Jose and we coordinated appointments when I was in town.”							
	“I am too healthy, you would better serve those that are newly injured.”							
	“I was already set up with my doctors so I felt comfortable continuing to see them.”							
	“I have had no complications since leaving the hospital.”							
(T) Do you have any additional comments/suggestions?	“I really appreciate the program and the iPad.”							
	“You guys do great work!”							
	“I loved the program and will continue to use TM visits.”							
	“The iPad made everything easier, even though I don’t have a lot of hand function, I can do everything on it. I use it all day, every day. You guys really nailed it with this program and obviously know what your patients need. I love it!”							

*Values are frequencies and (percentages)*.

*NA, not applicable; TM, telemedicine*.

**Table 3:** **Averages for quality of life (QoL) measures at baseline and 6 months (*n* = 8)**.

QoL measure	Baseline (mean ± SD)	Month 6 (mean ± SD)	*t*-Test (*p*-value)
Reintegration to Normal Living Index	70.1 ± 19.7	74.7 ± 21.8	0.79
Life Satisfaction Index A	25.4 ± 7.4	26.4 ± 8.2	0.37
Patient Health Questionnaire 9	6.8 ± 7.2	8.6 ± 6.1	0.59

### Quality of Life

Two patients did not complete the monthly follow-up interview or QoL measures at the 6-month time point; therefore, we have complete follow-up data of only eight participants. There were no statistically significant differences in outcome measures between the baseline and final follow-up (Table 3).

### Program Satisfaction Survey

Eight patients completed the Program Satisfaction Survey at the end of the 6-month period. Twenty questions were included in the survey ranging from individuals’ experience with the iPad device, the overall program, preferences for medical care, and anecdotal comments (Table 2). All participants reported positive experiences with the program. One hundred percent of program completers would recommend the program, and 100% of the participants who utilized TM reported that they would like to continue the program (Table 2, I and L). FaceTime on the iPad was not time consuming to utilize, patients felt comfortable about their privacy, and patients were satisfied with the quality of the visual image and audio sound during their TM visit(s) (Table 2, E–G). All patients reported that the iPad was easy for them to use and of the participants who utilized the adaptive equipment, all reported that the equipment was sufficient for their needs (Table 2, B and K). The iPad is a versatile device with many applications, so many participants were able to get a lot of use out of their iPad other than TM (Table 2, N and O). Most of the participants have Wi-Fi at home, which shows that they would be able to utilize an iPad from home without needing to pay for a data plan (Table 2, Q). The responses for top preference for medical care were mixed (Table 2, R). Anecdotal reports from the satisfaction survey indicated that this program was beneficial for the participants. A participant noted: “The iPad made everything easier, even though I don’t have a lot of hand function, I can do everything on it. I use it all day, every day. You guys really nailed it with this program and obviously know what your patients need” (Table 2, T).

## Discussion

This feasibility and proof of concept study showed the clinical viability of TM using iPads for individuals with SCI. Results indicate that participants were able to get connected and discuss medical issues with an SCI specialist that would have otherwise required cumbersome travel to a physician’s office. The type of interactions between clinicians and participants varied from generalized hospital follow-up and SCI primary care to specific questions on medications and coordination with subspecialty clinics. These results demonstrate the utility of TM beyond just that of pressure ulcer management. Additionally, these results demonstrate that this technology may be used by individuals within the home with the appropriate adaptive equipment, limiting the need for satellite clinics.

All participants sought care from a physician in-person at some point during the course of the six months, which had the highest number in terms of health-care utilization. This is not surprising as patients were not restricted from seeking in-person care as well as the need for potentially more complicated patients to receive care from other specialties such as urology. The trends found in the descriptive overview of the health-care utilization data show that more patients who did not use TM on a given month had a greater number of ER visits and hospitalizations reported. It is possible that having regular TM visits with an SCI specialist may be beneficial in preventing complications leading to ER visits or hospitalizations as the physician was able to address a variety of SCI-related concerns via FaceTime. However, it is also important to note that some ER visits and hospitalizations are unavoidable, and even if participants sought advice through TM they could have been advised to proceed in seeking emergency care.

Based on comments from the participants who did not have any FaceTime appointments, TM may be more beneficial for patients who are newly injured and recently discharged from inpatient rehabilitation, have medical complications to manage, and who are unable to travel long distances to have in-person visits with an SCI specialist; we hope to continually prioritize enrollment of individuals with SCI with these criteria in order to benefit more people and increase utilization.

The QoL outcome measures provide information on the participants’ return to their community and their QoL following their discharge from acute inpatient hospitalization. While the individual outcome measures did not show statistically significant differences between baseline and 6-month follow-up, this is not unexpected given the life changes that these patients face upon discharge from the hospital. Additionally, these findings are in line with previous studies showing no significant improvement in QoL within 6 months after hospital discharge (23).

The results from the Program Satisfaction Surveys demonstrate the ease of use and general acceptance of TM and iPad specifically as a tool for specialized SCI medical care in this patient population. The survey results were positive overall. Participants gave positive feedback regarding use of the iPad itself and the care they received. It is important to note that although five participants did not utilize FaceTime appointments for TM, all participants interacted with the program coordinator who served as a resource for addressing patients’ questions and a liaison for receiving advice from an SCI physiatrist or nurse. Additionally, the nature of the questions in the monthly follow-up questionnaire also acted as a check for general well-being. Although not all participants had TM through FaceTime, they were active participants in the TM program and gave valuable feedback regarding the implementation of this program. It is also important to note that the survey shows that most of the participants have access to a Wi-Fi connection at home. This is an important consideration for the implementation of a TM program such as this; even after the 6-month study is completed, participants were given the option to continue seeing a physiatrist through FaceTime. If participants have a Wi-Fi connection at home, they can continue their TM visits without the cost of a mobile data plan. Additionally, our facility has an online patient portal with a corresponding mobile application (MyHealth Online); although study participants are instructed to limit if not completely cease to contact the program coordinator as a resource once the 6-month study is over, participants can still have a direct connection to the physiatrist and Physical Medicine and Rehabilitation clinic by sending messages to providers through the online patient portal.

There were several challenges and lessons learned along the way. We occasionally encountered difficulties with connectivity and video reception, which may have been dependent on the location of the care provider and/or the participant. We noticed that older participants and/or care providers seemed to have more technical difficulties with the use of the iPad. Since the funding we received was to provide the iPads and the data plan only but not to fund the clinical visits themselves, we needed to obtain authorization for TM visits and we could only enroll patients who we knew ahead of time that we could obtain authorization for TM visits. Therefore, we are currently unable to provide TM care to patients with Medicare who do not live in the TM-certified areas.

### Limitations

Limitations of this study included the small population size; however, this will be addressed with the continuation of the study to enroll additional 110 participants; based on the successful implementation of this program, subsequent funding was received for the expansion and continued exploration of this project. The protocol for the expanded version of the study will differ very slightly in that the baseline RNLI questionnaire will be conducted at the 1-month follow-up rather than upon study enrollment; this is due to the fact that some of the questions on the RNLI are not applicable when asked while the participants are still in inpatient rehabilitation. Another limitation is that official records of medical care sought outside of the study institution were not available which may affect the accuracy of information reported by the participants; however, incorporating frequent monthly calls to patients from study staff minimized this limitation. Given the small sample size, our findings are descriptive and non-conclusive; therefore, future studies should have a greater sample size.

### Conclusion

Even though telehealth has been in existence for approximately 40 years, it is still not fully implemented as standard of care. This is the first known project using the iPad to provide TM in the SCI population and showed the feasibility of this intervention. These results provide evidence for using a tablet device like an iPad as an effective and efficient patient management tool to improve outcomes for individuals with SCI. Furthermore, these findings may be generalizable to other patient populations with other disabling conditions such as strokes, brain injury, and multiple sclerosis. TM in general can significantly improve patient care by improving convenience for the patients; the time and effort spent by SCI patients to travel for in-person clinic visits can be eliminated all while maintaining quality of care. Ultimately, when TM using any electronic device can be proven to be effective in the disabled patient population, then there will be an evidence-base to change health-care policy to allow for financial support and reimbursement for TM.

## Ethics Statement

This study was carried out in accordance with the approval of the Institutional Review Board’s Research and Human Subjects Review Committee of Santa Clara Valley Medical Center. All participants understood and gave written consent in accordance with the Declaration of Helsinki.

## Author Contributions

KS developed the SCiPad program including planning, designing, initiating, and implementing the program, screened potential participants, provided the clinical care of telemedicine to the participants, supervised the project overall, and prepared this manuscript along with her coauthors. SS is the succeeding program coordinator for this project; SS performed data analysis, preparation of figures/tables, literature searches, and manuscript preparation. KS and SS contributed to the manuscript as cofirst authors. EL performed background literature searches, data analysis, and manuscript preparation. LI supervised Rehabilitation Research Center staff such as SS and assisted with manuscript preparation.

## Conflict of Interest Statement

The authors declare that the research was conducted in the absence of any commercial or financial relationships that could be construed as a potential conflict of interest.
